# Canadian health library cuts and closures: what CHLA/ABSC is doing, and what you can do too

**DOI:** 10.29173/jchla29816

**Published:** 2024-12-01

**Authors:** Amanda Ross-White, Jeff Mason

**Affiliations:** 1 Health Sciences Librarian, Bracken Health Sciences Library, Queen’s University, Kingston, ON; President, CHLA-ABSC; 2 Health Sciences Librarian: Innovation and Entrepreneurship, Health Sciences Library, McMaster University, Hamilton, ON

## Advocacy

Demonstrating the value of Canadian health libraries and library professionals is one of the four pillars of the Canadian Health Libraries Association / L’Association des bibliothèques de la santé du Canada (CHLA/ABSC)’s 2023-2025 strategic plan [1]. Advocacy, as the French word for lawyer, *avocat*, brings out so prominently, is to speak in defense of someone when they are unable to do so themselves.

CHLA/ABSC has been very active in recent months, advocating for its members and the profession [2]. There are many ways to advocate. Each method has value and can be considered by CHLA/ABSC members facing uncertainty in challenging times. Here are some of the ways CHLA/ABSC has approached advocacy that you can do too.

## Letter writing

Writing a letter is one of the first ways to engage with decision makers and let individuals and groups affected by an action or event where advocacy is called for, know about your concerns. While letter writing may seem like a small step, it can be a vital tool for forcing a response. In the wake of library worker terminations and information services restructuring at Canada’s Drug Agency (CDA, formerly CADTH) in the spring of 2024, letter writing was CHLA/ABSC’s first course of action. CHLA/ABSC sent letters to CDA’s funders (federal, provincial, and territorial ministers of health), its board of directors, and opposition party shadow ministers and critics [3]. Because CDA is funded by the federal, provincial, and territorial governments collectively, CHLA/ABSC expected a response from at least some of the recipients, and it worked. CHLA/ABSC received responses from the Health Minister of Nova Scotia, the Health Minister of Manitoba, and the shadow minister of Alberta [2].

## Petitions

Alongside a letter writing campaign, CHLA/ABSC started a petition on www.change.org in support of the CDA library [4]. A second www.change.org petition [5] launched following the announcement of the College of Physicians and Surgeons of British Columbia (CPSBC) library’s closure after 100 years of service [6,7]. While CDA is a government-funded agency and on some level is responsive to the public, CPSBC is a private member-led organization. They are under no obligation to respond to CHLA/ABSC petitions, although they should be responsive to their members. Petitions are beneficial for demonstrating the level of support an advocacy campaign has behind it. At the time of writing, the CPSBC petition [5] has over 11,000 signatures and the CDA petition [4] over 2000. The number of supporters for a petition, however, is not a petition’s only strength. Petitions are also useful for finding out who your supporters are. Seeing influential names sign a CHLA/ABSC petition, people like Dr. Andrea Tricco [8], Dr. David Moher [9] and Dr. Jeremy Grimshaw [10], help CHLA/ABSC understand who is engaged in the debate and who may be an ally in adding additional pressure for the cause.

## Media exposure

Health libraries are niche organizations. There are only a few hundred library and information workers employed in health libraries across Canada. For this reason, it can be hard to get the attention of media outlets. Yet engaging with the media can be a useful advocacy strategy to demonstrate the value of health libraries and health information workers to new audiences. A skilled reporter can help frame a complex issue such as the closure of a hospital library in a way that helps listeners, readers, and viewers connect with an event or an issue and make sense of its potential impacts, such as Joanna Frketich's recent article on the closure of the library at St Joseph’s Healthcare in Hamilton, ON [11]. With the CPSBC campaign, CHLA/ABSC engaged local media by sending them copies of advocacy letters written by CHLA/ABSC and had www.change.org contact them when CHLA/ABSC’s petition started to take off. Through its role of informing the public and holding our leaders accountable, the media can indirectly increase pressure on decision makers who pay attention to what their constituents are talking about. Working with the media is not for everyone but it is a skill that can be learned. Not everyone is comfortable speaking to journalists live, but responding to questions in writing allows more time to research and reflect on your answers. Pre-recorded interviews may also be an option. Some things to remember are that while the media work with deadlines, they are rarely instantaneous. You can ask them to call back and send questions in advance. You can prepare and practice beforehand. If you work at an academic library, your employer likely has media training opportunities to help prepare you for success.

## Everyday advocacy

While not every closure or restructuring can be anticipated, there are strategies we, as individuals, colleagues, and a profession, can use to continuously and concretely demonstrate and communicate our value [12].

### Prepare and practice your elevator speech and value talking points

You never know when you might find yourself with the opportunity to talk with the CEO and you may only have a moment to get your message across.

### Know your value

High use of your services does not always equate to value, and usage data does not fully communicate the impact the library has on the organization’s values and mission. If your library is part of a health or social services organization like a hospital or public health unit, you may also want to become familiar with CHLA/ABSC’s standards for these types of institutions [13].

### Use your academic freedom

Many librarians working in higher education are afforded the freedom to speak out on causes that our colleagues in the public and private sector are not. As such, many academic librarians can act as the voice and face for our colleagues and profession in times of need. For example, prior to engaging with the media during its CDA and CPSBC campaigns, the CHLA/ABSC Board discussed who was best suited to represent the association and its members. The CHLA/ABSC President, Amanda Ross-White, had previous media experience and an academic position with protected academic freedom, so she felt comfortable volunteering.

### Network, network, network

Keep cultivating your networks so that in times of need you can quickly identify champions and reach out to supporters who hold influence with your organization.

And although letter writing, petitions, and working with the media are all strong approaches for advocacy during challenging times, the best time to advocate is *before* cuts and closures happen.
